# Pharmacokinetics and Tissue Distribution of Palmatine after Single Intramuscular Injection in Partridge Shank Chickens

**DOI:** 10.1002/vms3.70416

**Published:** 2025-06-19

**Authors:** Chaoxi Chen, Yue Bu Mo A‐ga, Xiuhua Kuang, Ye Li, Rong Xiang, Jun Liang

**Affiliations:** ^1^ College of Animal and Veterinary Sciences, Southwest Minzu University Chengdu China; ^2^ College of Veterinary Medicine, Henan University of Animal Husbandry and Economy Zhengzhou China; ^3^ Technology Center of Dalian Customs Dalian China; ^4^ Institute of Animal Health, Guangdong Academy of Agricultural Sciences/Scientific Observation and Experiment Station of Veterinary Drugs and Diagnostic Techniques of Guangdong Province Guangzhou China; ^5^ Zhengzhou Inspection and Testing Center of Product Quality Zhengzhou China

**Keywords:** palmatine, partridge shank chickens, pharmacokinetics, tissue distribution

## Abstract

Palmatine is an isoquinoline alkaloid isolated from many medicinal plants with diverse pharmacological activities, with potential for the treatment of chronic metabolic disorders and infectious diseases. In the present study, a highly rapid, simple, and sensitive ultra‐performance liquid chromatography coupled with an ultra‐violet (**UPLC‐UV**) method was developed and validated for the pharmacokinetics and tissue distribution of palmatine following single intramuscular injection to Partridge Shank chickens. The process was sensitive, with a lower limit of quantification and good linearity over the range of 0.005‐10 µg/mL for UPLC‐UV in all biological matrices. Following a single intramuscular dose of 5 mg/kg b.w, palmatine was absorbed quickly and reached peak concentration (0.24 µg/mL) at 0.19 h and was still present in plasma at 12 h with a concentration of 0.011 µg/mL. The CL/F and Vd/F were 10.43 L·h^−1^·kg ^−1^ and 56.69 L/kg, respectively. The *C*_max_ values of palmatine in tissues decreased as follows: kidneys > liver > spleen > heart > lung > muscles, and were almost completely cleared from lungs 26 h after administration and were not detectable or significantly reduced in other tissues after 48 h. The results can provide a basis for exploring the therapeutic potential of palmatine and looking for new ways of wide clinical applications in poultry farming.

## Graphical abstract

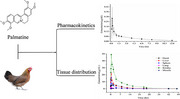

## 1 Introduction

With the rise of bacterial infections and the emergence of multidrug‐resistant bacterial strains in humans and animals, extensive and long‐time exposure to synthetic drugs can lead to adverse effects (Parmanik et al. 2022). Herbal medicine is widely used for the prevention and treatment of infectious and chronic diseases due to its low toxicity, cost‐effectiveness and promising therapeutic efficacy. Many researchers have focused on Chinese herbal medicines and/or their active ingredients for non‐antibiotic therapeutics for infectious diseases and chronic diseases (Zhang et al. 2013; Tian et al. 2023).

Palmatine is an isoquinoline alkaloid isolated from many medicinal plants such as *Coptis chinensis* Franch., *Phellodendron amurense* Rupr., *Fibraurea recisa* Pierre. and *Mahonia aquifolium*, exhibiting diverse pharmacological activities in the treatment of jaundice, dysentery, hypertension and infections caused by bacteria, viruses and fungi (Abidi et al. 2006; Wu et al. 2008; Zhang et al. 2011). Many medicinal plants and natural drug formulations containing palmatine have been widely used in animal breeding, but detailed information about the use of palmatine in food animals remains relatively inadequate. Aghayan has also reported that palmatine could reverse multidrug resistance by inhibiting MexAB‐OprM efflux pumps (Aghayan et al. 2017). Currently, the simultaneous detection, pharmacology, toxicity and pharmacokinetics of palmatine and other isoquinoline alkaloids have been reported in humans, canines, rabbits and rats. However, the tissue distribution study has been limited to rats and most of the studies mainly focus on developing quantitative methods for palmatine and other protoberberine alkaloids, including berberine, coptisine and jatrorrhizine, in various crude drugs, decoctions and preparations (Ma et al. 2009; Liu et al. 2011; Vrba et al. 2015).

As an important local chicken breed in China, Partridge Shank chickens embody the basic characteristics of native chickens and have many advantages, such as strong environmental adaptability, rapid growth performance and high reproductivity. Meanwhile, coupled with the unique flavour characteristics and nutritional quality of meat products, the market‐oriented attention of Partridge Shank chickens is attracting (Xi et al. 2014; Chen et al. 2018; Zhao et al. 2019). To the author's knowledge, palmatine used to treat bacterial infections in Partridge Shank chickens, has not been studied until now. Collectively, considering the potential for its application in the animal breeding industry, the pharmacokinetics and tissue distribution of palmatine after intramuscular administration are within the scope of the present research, exploring the therapeutic potential of this compound and looking for new ways of wide clinical applications in poultry farming.

## 2 Materials and Method

### 2.1 Animals

One hundred and seventy clinically healthy Partridge Shank chickens (body weight of 1.5 ± 0.18 kg, 90 days old, male to female ratio was 1:1) were employed and provided by a poultry farm in Huiji district of Zhengzhou city, central China's Henan province. All the animals were housed in an environmentally controlled room maintained at 25 ± 1°C with a relative humidity of 55 ± 10% and a 12 h light/dark cycle. They were fed an antibacterial‐free diet and had free access to water.

The experimental procedures were performed in line with the guidelines of the Laboratory Animal Management Statute of the College of Veterinary Medicine, Henan Agricultural University and approved by the Animal Ethics Research Committee of the Faculty of Henan Agricultural University (HNAU‐202101061). Before inclusion in the study, each animal was deemed healthy based on health history and physical examination.

### 2.2 Chemicals and Reagents

Palmatine hydrochloride (purity > 98.0%) and berberine hydrochloride (purity > 98.7%) standards were purchased from the China Institute of Veterinary Drug Control (Beijing, China), and berberine hydrochloride was used as the internal standard (**IS**) for analysis. HPLC‐grade acetonitrile and methanol were commercially obtained from Sigma‐Aldrich (St. Louis, MO, USA). Purified water was produced using the Milli‐Q water purification system and all the other chemicals and reagents used during the extraction and analysis were of analytical grade.

### 2.3 Experimental Design and Analytical Methods

#### 2.3.1 Dosing Formulations

Palmatine solution for intramuscular injection was prepared freshly by dissolving an appropriate amount of palmatine hydrochloride in 0.9% sterile sodium chloride solution and passing through a 0.22 µm filter. A dose of 5 mg/kg b.w was used for pharmacokinetics and tissue distribution.

### 2.4 Pharmacokinetics Study

Twenty Partridge Shank chickens with a 1:1 ratio of male and female were weighed and the left pectoral muscle was the ideal injection point for a single dose of palmatine hydrochloride. Blood samples (0.8 mL) were taken from the lateral wing vein of each bird with heparinised syringes at 0, 5, 10, 15, 30 min, 1, 1.5, 2, 4, 6, 8, 12 and 24 h after administration. Plasma was separated as described in the sample preparation section.

### 2.5 Tissue Distribution of Palmatine in Partridge Shank Chickens

One hundred and fifty Partridge Shank chickens (75 female and 75 male) were housed in separate chicken coops. Ten birds were sacrificed by air embolism via lateral wing vein at predetermined time points 0, 5, 10, 20, 30 min, 1, 2, 4, 8, 12, 16, 24, 26, 42 and 48 h after receiving a single intramuscular dose of palmatine hydrochloride. Consequently, the tissue samples (heart, liver, spleen, lung, kidney and muscle) were taken each time from the slaughtered birds and washed with pretreated ice‐cold 0.9% sodium chloride solution. The samples were accurately weighed and subjected to the processing as described in the sample preparation section and the tissue distribution of palmatine was determined.

### 2.6 Ultra‐Performance Liquid Chromatography‐Ultraviolet(UPLC‐UV)Conditions

Chromatographic separations were performed on an ACQUITY UPLC I‐Class system with reversed‐phase C_18_ columns maintained at a temperature of 20°C and a flow rate of 0.25 mL/min. The injection volume was 5 µL and the wavelength was 345 nm. For plasma, a reversed‐phase C_18_ column (BEH C_18_, 1.7 µm, 2.1 mm × 150 mm, Waters, USA) was selected, and a reversed‐phase C_18_ column (BEH C_18_, 1.7 µm, 2.1 mm × 100 mm, Waters, USA) was used for tissues (muscle, heart, liver, spleen, lung and kidney). The mobile phase was comprised of a mixture of 0.4% phosphate buffer solution (pH 3.5) and acetonitrile at a ratio of 68:32 (v/v).

### 2.7 Mixed Stock Solution and Working Standard Solution Preparation

A mixed stock solution of palmatine and berberine at 100 µg/mL was prepared with the mobile phase. Finally, working standard solutions for calibration standards (**CS**) and quality control (**QC**) in a concentration range of 0.005, 0.01, 0.02, 0.05, 0.1, 0.2, 0.5, 1, 2, 5 and 10 µg/mL were prepared in mobile phase by serial dilution method. All of the solutions were stored in a refrigerator at ‐20°C until analysis.

### 2.8 Preparation of Calibrators and Quality Control Samples

CS and QC samples were prepared freshly by spiking the appropriate amount of working standard solution into 100 µL of blank plasma and tissue suspension. CS samples were prepared at 11 concentrations of 0.005‐10 µg/mL and QC samples were at 3 levels of 0.01 (lower limit of quantification, LLOQ), 0.1 (low QC), and 1 (middle QC) µg/mL. Five duplicates of the quality control plasma and tissue at three concentration levels were prepared and used to determine the linearity, recoveries, intra‐day and inter‐day precision and accuracy of the analytical method. The procedure was repeated three times within the same day and over three different days to obtain intra‐day and inter‐day run precision and accuracy, respectively.

### 2.9 Sample Extraction Procedure for UPLC‐UV

The extraction of palmatine from plasma was performed by a simple protein precipitation technique and performed according to Huang et al. (2007) with minor modifications. Briefly, the blood samples were centrifuged at 16,000 ×g for 5 min at 4°C, 1.5 mL of chloroform containing 15 mg/mL of trichloroacetic acid (**TCA**) was added to 0.5 mL plasma in a 5 mL stoppered test tube and followed by vigorous vortex‐mixing for 20 min and ultrasonic treatment for 10 min. The supernatant was transferred to a new test tube after centrifugation at 16,000 ×g for 10 min. The procedure was repeated and the collected supernatant was evaporated to dryness under a gentle stream of nitrogen in a water bath at 50°C. The residue was redissolved in 0.5 mL mobile phase, vortexed for 5 min and exposed to ultrasound treatment for 10 min. Finally, 50 µL of water‐saturated n‐hexane was added, after vortex mixing for 5 min, the lower supernatant was centrifuged at 16,000 ×g for 10 min and filtered through a 0.22 µm filter.

For tissues (heart, liver, spleen, lung, kidney and muscle), the whole tissues were collected and washed with a pretreated ice‐cold 0.9% sodium chloride solution. Then, 1 g of minced tissue was weighed, 1 mL distilled water and 1.5 mL chloroform containing 15 mg/mL TCA were added, followed by vigorous vortex mixing for 20 min and ultrasonic treatment for 10 min. The supernatant was transferred to a new test tube after centrifugation at 16,000 ×g for 10 min. The procedure was repeated three times and the collected supernatant was evaporated to dryness under a gentle stream of nitrogen in a water bath at 50°C and then the residue was redissolved in 1 mL mobile phase and 0.6 mL of water‐saturated n‐hexane, shaken vigorously for 10 min and centrifuged at 16,000 ×g for 20 min. Finally, the lower supernatant was filtered through a 0.22 µm filter.

### 2.10 Method Validation

Selectivity was investigated by analysng blank plasma and tissues from 5 individual Partridge Shank chickens that were not administered palmatine hydrochloride and comparing them with the corresponding spiked biological matrices at the LLOQ level or the samples collected after intramuscular administration. There should be no interference from the endogenous substances at the retention times of both palmatine hydrochloride and berberine hydrochloride using the pretreatment procedure and chromatographic conditions.

The calibration curve was acquired by comparing the peak area ratio of palmatine hydrochloride to IS by weighted least‐squares linear regression. The limits of detection (**LOD**) and quantification (**LOQ**) were defined as the lowest concentration of the calibration curve with an S/N ratio greater than 3 and 10, respectively. The acceptable accuracy (RE, %; ± 20%) and precision (RSD, %; ≤ 20) were required.

Accuracy and precision were determined in all biological matrices at QC concentrations (LOQ, LQC, and MQC; n = 5 each) using different analytical batches on the same day and three consecutive days. The acceptance criteria for intra‐ and inter‐day precision (RSD, %) were required to be ≤ 15% except for LOQ, whose precision should be ≤ 20% and accuracy (RE, %) was limited to ± 15% except for LOQ (± 20%).

### 2.11 Software and Data Processing

Pharmacokinetic calculations (noncompartmental analysis) were performed by Model 201 (extravascular input, plasma) in WinNonlin 5.2 (Pharsight Corp., Cary, NC, USA). The descriptive statistics were calculated using Microsoft Excel.

## 3 Results

### 3.1 Optimisation of UPLC‐UV Conditions

**FIGURE 1:**
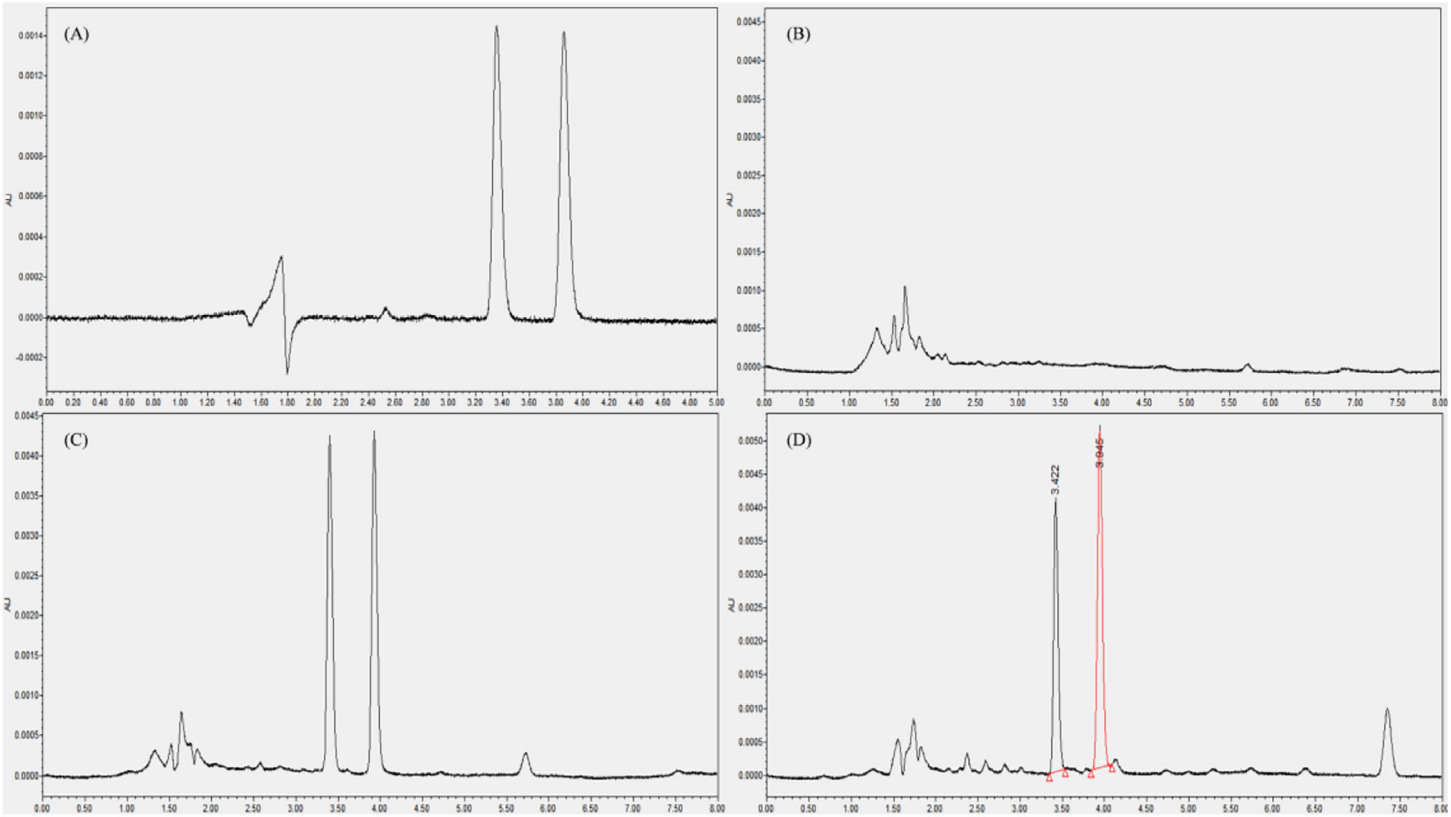
UPLC‐UV chromatograms of palmatine and berberine in plasma. (A) Mixed standard solution of palmatine and berberine, (B) blank plasma, (C) plasma spiked with palmatine and berberine and (D) palmatine and berberine in plasma profile after administration.

**FIGURE 2:**
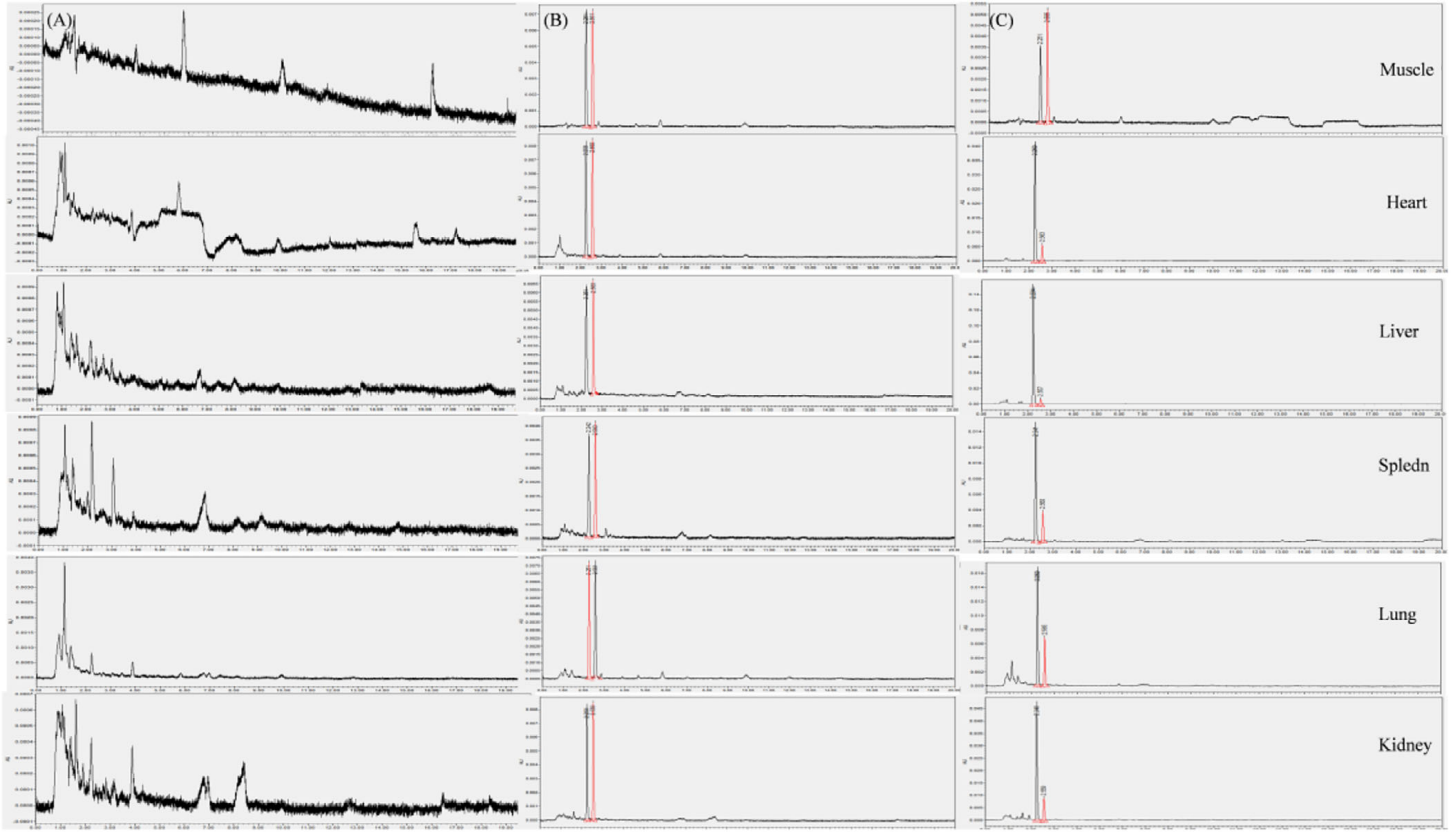
UPLC‐UV chromatograms of palmatine and berberine in tissues (muscle, heart, liver, spleen, lung and kidney). (A) Blank tissues, (B) tissues spiked with palmatine and berberine and (C) palmatine and berberine in tissue profiles at different time points.

The UPLC‐UV chromatograms of palmatine and berberine in plasma and tissues (muscle, heart, liver, spleen, lung and kidney) are shown in Figures 1, 2. The retention times (**RT**) of palmatine and berberine were 3.42 min and 3.94 min in plasma, 2.25 min and 2.56 min in tissues, respectively.

For UPLC‐UV analysis, an acetonitrile‐phosphate buffer system was selected to obtain chromatograms with good resolution and symmetric peak shapes in palmatine and berberine. It was found that 0.4% aqueous phosphoric acid‐acetonitrile (68:32, v/v) enhanced the sensitivity with small deviations in inter‐day and intra‐day RT, overcoming the interference of biological matrices and improving peak symmetry in UPLC‐UV analysis. During method development, a chromatogram of good peak shape and resolution was obtained for plasma and tissues using a mobile phase according to the method validation procedure, respectively.

### 3.2 Method Validation

The developed UPLC‐UV method was selective, with no significant endogenous interference from plasma and tissues observed at the retention times of palmatine and IS (± 2.5%), respectively. The regression equations, LOD, and LOQ for the UPLC‐UV method in Partridge Shank chicken plasma and tissues are listed in Table 1. All validation parameters were calculated using the relative peak area for palmatine, the calibration curves showed good linearity over the concentration ranges of 1–1000 ng/mL. The calibration curves were linear over the calibration range for palmatine, resulting in R^2^ values between 0.9991 and 0.9992. The determined LOD and LOQ for palmatine were in the 2–10 ng/mL and 5–20 ng/mL ranges for different matrices, respectively.

**TABLE 1:** The calibration curves, LOD and LOQ of palmatine and berberine in Partridge Shank chicken plasma and tissues (µg/mL).

	Palmatine				Berberine			
Matrix	Calibration curves	R^2^	LOD	LOQ	Calibration curves	R^2^	LOD	LOQ
Plasma	Y = 52896X+414.31	0.9991	0.002	0.005	Y = 60145X‐535.42	0.9992	0.002	0.005
Muscle	Y = 48740X+516.26	0.9991	0.005	0.010	Y = 54973X‐352.83	0.9992	0.005	0.010
Heart	Y = 48883X+359.53	0.9991	0.005	0.010	Y = 560533X‐567.9	0.9991	0.005	0.010
Liver	Y = 47765X+377.78	0.9991	0.010	0.020	Y = 54318X‐554.17	0.9992	0.010	0.020
Spleen	Y = 48354X+305.43	0.9992	0.010	0.020	Y = 54833X‐511.6	0.9991	0.010	0.020
Lung	Y = 47689X+428.3	0.9991	0.005	0.010	Y = 54430X‐488.05	0.9991	0.005	0.010
Kidney	Y = 48276X+373.41	0.9991	0.010	0.020	Y = 54716X‐513.71	0.9992	0.010	0.020

The intra‐day and inter‐day precision of palmatine and berberine in standard solution is presented in Table 2. The intra‐day and inter‐day precision (RSD, %) values were both < 10% for UPLC‐UV. The results showed that all the RSD values were in the acceptable ranges, indicating that the established UPLC‐UV method was reliable and reproducible for the determination of palmatine and berberine in plasma and tissues.

**TABLE 2:** The intra‐day and inter‐day precision (RSD, %) of palmatine and berberine in standard solution.

Nominal concentration (µg/mL)	Palmatine		Berberine	
	Intra‐day	Inter‐day	Intra‐day	Inter‐day
0.010	0.54	0.31	0.43	0.28
0.100	0.41	0.20	0.24	0.19
1.000	0.25	0.18	0.31	0.15

The absolute recovery of palmatine and berberine in plasma and tissues is presented in Table 3. With a minimum of 3 concentration levels and 3 replicates per level (9 determinations), the absolute recovery of palmatine and berberine was > 78.89% and > 78.74% in the UPLC‐UV method. The above results indicate that the extraction and purification method in this study is suitable and reliable for palmatine and berberine in plasma and tissues.

**TABLE 3:** Absolute recovery and coefficient of variation (CV) of palmatine and berberine in Partridge Shank chicken plasma and tissues (n = 10).

Matrix	Nominal concentration (µg/mL)	Palmatine		Berberine	
		Mean (%)	CV (%)	Mean (%)	CV (%)
Plasma	0.01	85.86	3.06	84.25	3.85
	0.10	91.07	2.22	90.81	2.00
	1.00	89.34	2.41	89.17	2.59
Muscle	0.05	81.37	3.85	81.03	3.73
	0.20	85.29	2.54	84.94	2.41
	1.00	82.36	3.21	81.39	2.80
Heart	0.05	80.45	4.24	81.05	4.08
	0.20	83.03	2.75	83.19	2.62
	1.00	82.55	3.44	82.92	3.29
Liver	0.05	78.89	4.65	78.74	4.51
	0.20	81.54	2.89	80.81	2.77
	1.00	80.67	3.69	80.36	3.33
Spleen	0.05	79.92	4.05	79.20	4.27
	0.20	81.25	2.79	81.73	2.64
	1.00	81.64	2.88	81.12	2.79
Lung	0.05	80.75	4.31	80.64	4.14
	0.20	82.03	3.35	81.17	3.22
	1.00	80.56	3.56	80.59	4.21
Kidney	0.05	78.91	4.25	79.22	4.13
	0.20	82.37	3.29	82.13	3.08
	1.00	81.53	3.48	80.96	3.77

### 3.3 Pharmacokinetics of Palmatine

**FIGURE 3:**
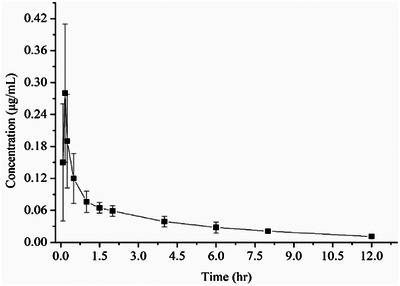
Mean plasma concentration‐time curves for palmatine in Partridge Shank chickens after intramuscular administration at 5 mg/kg b.w. Data presents mean ± SD values for 10 subjects.

The developed and validated UPLC‐UV method was successfully applied for the determination of palmatine pharmacokinetics. After a single intramuscular injection of 5 mg/kg b.w to Partridge Shank chickens, the mean plasma concentration versus time profiles of palmatine are illustrated in Figure 3, and the major non‐compartmental pharmacokinetic parameters from model analysis are listed in Table 4. The profile revealed that after intramuscular administration, palmatine was absorbed quickly and reached peak concentration (0.24 µg/mL) at 0.19 h. Thereafter, the drug concentration in serum declined gradually. Palmatine was still present in plasma at 12 h and the concentration was 0.011 µg/mL (over the LOQ). However, palmatine could not be detected after 16 h. The elimination half‐life (*T*_1/2β_) and MRT_0→∞_ were 3.98 h and 5.06 h, respectively. In a word, the characteristic pharmacokinetic properties of palmatine include fast intramuscular absorption, low clearance and extensive distribution into tissues.

**TABLE 4:** Non‐compartmental pharmacokinetic parameters of palmatine in Partridge Shank chickens following intramuscular injection at 5.0 mg/kg b.w (n = 10).

Parameters	Units	I.M.
*T*_1/2β_	h	3.98 ± 0.88
*T*_max_	h	0.19 ± 0.04
*C*_max_	µg/mL	0.24 ± 0.12
CL/F	L·h^−1^·kg^−1^	10.43 ± 4.74
Vd/F	L/kg	56.69 ± 13.95
AUC_0→∞_	µg·h/mL	0.53 ± 0.12
AUMC_0→∞_	µg·h·h/mL	2.72 ± 0.82
MRT_0→∞_	h	5.06 ± 0.91

Abbreviations: AUC_0→∞_, the area under the serum concentration‐time curve from zero to infinity; AUMC_(0→∞)_, the area under the first‐moment of the concentration‐time curve with extrapolation to infinity; CL, body clearance; CL/F, body clearance corrected for bioavailability; *C*_max_, the maximum concentration; MRT_0→∞_, the time required to eliminate 63.2% of the drug; *T*_1/2β_, the elimination half‐life; T_max_, the time to peak concentration; Vd, volume of distribution; Vd/F, volume of distribution corrected for bioavailability.“ / ” means not calculated.

### 3.4 Tissue Distribution of Palmatine

**FIGURE 4:**
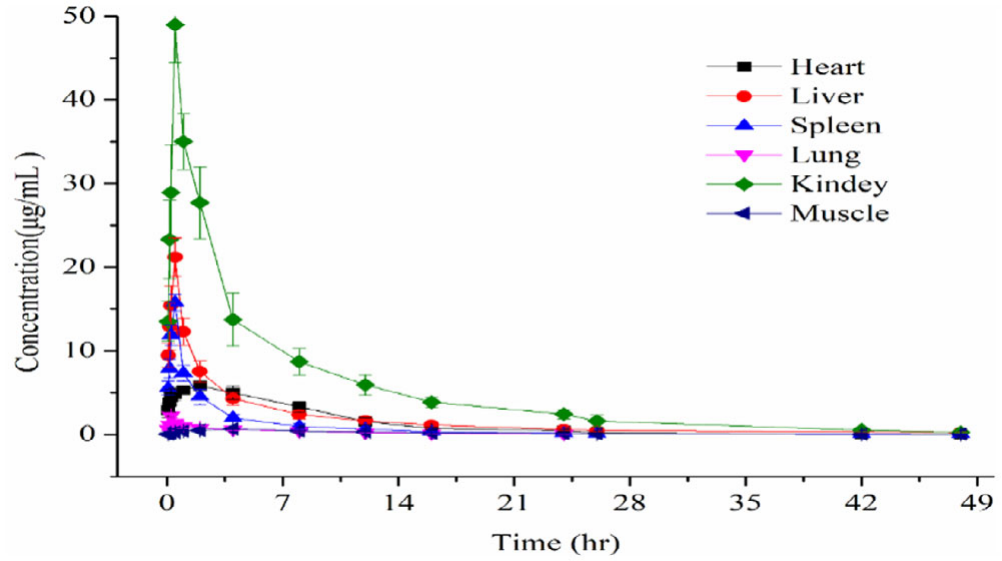
Biodistribution of palmatine in heart, liver, spleen, lung, kidney and muscle following intramuscular administration at 5 mg/kg b.w. Data presents mean ± S.D. values for 10 subjects.

The concentrations of palmatine in various tissues of Partridge Shank chickens are represented in Figure 4 and Table 5. A higher concentration of palmatine was found in the kidney (48.99 ± 4.55 µg/mL), liver (21.21 ± 2.29 µg/mL), and spleen (15.80 ± 1.0 µg/mL) and a lower concentration in the heart (5.85 ± 0.45 µg/mL), lung (2.29 ± 0.30 µg/mL), and muscle (0.69 ± 0.07 µg/mL). The reason was the higher accumulation of palmatine and its possible metabolism and excretion pathways by these organs. Palmatine was almost completely cleared from the lungs 26 h after administration and was not detectable or significantly reduced in other tissues after 48 h. The mean *C*_max_ values of palmatine for the spleen, liver and kidney were obtained at about 0.5 h after intravenous administration. For lung, heart, and muscle, the mean *C*_max_ values of palmatine were obtained at 30 min, 2 h and 4 h, respectively. The mean *C*_max_ values of palmatine in tissues decreased as follows: kidney > liver > spleen > heart > lung > muscle. The elimination half‐lives (*T*_1/2β_) for palmatine in tissues were over 6 h and their descending order was: spleen > liver > muscles > kidney > heart > lung. The AUC_0→∞_ values for all the tissues decreased after administration and the order was as follows: kidney > liver > heart > spleen > muscle > lung.

**TABLE 5:** The concentration of palmatine in the heart, liver, spleen, lung, kidney and muscle following intramuscular administration at a dose of 5 mg/kg b.w (µg/mL).

Time (h)	Muscle	Heart	Liver	Spleen	Lung	Kidney
0.083	0.06 ± 0.02	2.92 ± 0.88	9.46 ± 1.41	5.57 ± 0.79	0.68 ± 0.16	13.54 ± 2.36
0.17	0.16 ± 0.04	3.88 ± 1.12	12.92 ± 2.77	7.92 ± 1.12	1.19 ± 0.26	23.33 ± 4.67
0.25	0.21 ± 0.05	4.48 ± 0.94	15.43 ± 2.37	11.90 ± 1.23	2.29 ± 0.30	28.95 ± 5.72
0.5	0.31 ± 0.06	4.86 ± 0.74	21.21 ± 2.29	15.80 ± 1.0	1.43 ± 0.31	48.99 ± 4.55
1	0.43 ± 0.08	5.31 ± 0.45	12.31 ± 1.62	7.34 ± 0.95	1.03 ± 0.26	35.01 ± 3.33
2	0.53 ± 0.06	5.85 ± 0.45	7.56 ± 1.26	4.53 ± 0.91	0.81 ± 0.22	27.72 ± 4.28
4	0.69 ± 0.07	5.00 ± 0.80	4.31 ± 0.79	2.02 ± 0.45	0.63 ± 0.15	13.74 ± 3.18
8	0.47 ± 0.05	3.34 ± 0.64	2.41 ± 0.49	1.00 ± 0.29	0.43 ± 0.11	8.72 ± 1.60
12	0.35 ± 0.06	1.57 ± 0.42	1.68 ± 0.38	0.69 ± 0.13	0.24 ± 0.06	5.96 ± 1.22
16	0.21 ± 0.03	0.76 ± 0.19	1.17 ± 0.29	0.34 ± 0.06	0.14 ± 0.05	3.88 ± 0.64
24	0.16 ± 0.03	0.48 ± 0.09	0.64 ± 0.11	0.18 ± 0.02	0.08 ± 0.03	2.45 ± 0.54
26	0.11 ± 0.04	0.21 ± 0.09	0.48 ± 0.07	0.13 ± 0.03	/	1.65 ± 0.74
42	0.07 ± 0.02	0.07 ± 0.03	0.36 ± 0.07	0.08 ± 0.01	/	0.56 ± 0.15
48	0.05 ± 0.01	0.02 ± 0.01	0.26 ± 0.03	0.07 ± 0.01	/	0.29 ± 0.09

## 4 Discussion

Palmatine is an isoquinoline alkaloid with extensive biological activities such as antibacterial, antifungal, and antiviral activity. Many studies have focused on the quantitative methods to determine the content of palmatine, including HPLC (Long et al. 2019) and LC‐MS (Lu et al. 2006) and its pharmacokinetics in different animals. In this study, the UPLC‐UV method was established for detecting palmatine in plasma and tissue (muscle, heart, liver, spleen, lung and kidney). Meanwhile, the recovery, precision and sensitivity of the method were improved. Berberine was used as the internal standard, the characteristic absorption peaks of UV palmatine and berberine were similar at 227 nm, 270 nm and 345 nm and the detection wavelength of 345 nm was selected since the biological impact of shorter wavelength ultraviolet radiation (Nishigori et al. 2023). We also attempted to develop a UPLC‐MS/MS method for the quantification of palmatine using berberine as an internal standard. However, it was found that palmatine was demethylated to form berberine, resulting in severe interference. Furthermore, the commercial product of deuterated palmatine is not yet available, so a qualitative analysis of palmatine by UPLC‐MS/MS was not developed. Concerning the serious ion suppression and matrix effect in the UPLC‐MS/MS analyses for palmatine could not meet the requirements, UPLC‐UV was used in the pharmacokinetic and tissue distribution in this study, exhibiting the advantages of simple sample preparation, high sensitivity and selectivity.

Organic solvents, such as methanol, trichloromethane, acetonitrile and acetone, were compared in developing the protein precipitation procedure along with the evaluations for matrix effect, recovery efficiency and reproducibility. As an ion‐pairing reagent, TCA was selected for biological matrices for protein precipitant in this study, which yielded high recovery rates without interference, low RSD of inter‐day run and intra‐day run precision and accuracy following the report (Zhang et al. 2008).

Many alkaloids showed poor absorption and low oral bioavailability, affected by factors including poor solubility, slow dissolution rate, first‐pass effect and so on. Our pre‐experimental results showed that palmatine was not absorbed after a single dose of 10 mg/kg via intragastric gavage, the clearance kinetics of palmatine werew rapid and the plasma concentration decreased by approximately half compared to the initial concentration at 0.25 h and could not be detected at 6 h after intravenous injection (data not shown), accounting for its limited absorption after oral administration. However, after intramuscular administration at a single dose of 5 mg/kg b.w, palmatine was quickly absorbed, well distributed and slowly eliminated, resulting in the peak plasma concentration at about 0.19 h and could not be detected after 16 h. The parameter Vd/F was 56.69 L/kg, showing similar absorption and distribution characteristics in canines (Huang et al. 2007).

Many studies have elucidated the pharmacokinetics and tissue distribution of palmatine in humans, rabbits, dogs and rats, there exist a double‐peak phenomenon (Shia et al. 2011). Although the dosage regimen used in this study may offer a good option for the treatment of bacterial infections in Partridge Shank chickens, it is necessary to note that palmatine is present in many heat‐clearing and detoxicating herbs and is an essential component of Traditional Chinese Medicine (TCM) formulas used in human medicine; therefore, it is suggested that vigilance and veterinary supervision are needed when administering palmatine to food‐producing animals.

The liver CYP450 superfamily is important and plays a crucial role in the metabolism of various exogenous and endogenous substances. Pharmacokinetic studies have demonstrated that glucuronidation and sulfation are the main metabolic pathways of palmatine, sulfotransferases and UDP glucuronic acid transferases play an important role in phase II metabolic reactions in different species (Long et al. 2019). Up to now, few studies have focused on the metabolism and excretion of palmatine in food animals. Our ongoing work will focus on the expected metabolic pathways (e.g., glucuronidation and sulfation) of palmatine in Partridge Shank chickens by HPLC‐MS/MS coupled with other methods such as reference‐based, common approach, identification of gene sequence and sequence homology. Meanwhile, the 7‐in‐1 substrate cocktail concurrent administration to simultaneously assess the direct induction and/or inhibition of palmatine on Partridge Shank chickens provides a reference to assess CYP450 induction and/or inhibition and time‐dependent inhibition of isolated plant‐derived natural products. However, current research on the tissue distribution of palmatine is very limited and few researchers have dealt with its metabolism and excretion. Based on the pharmacokinetics and tissue distribution of palmatine in Partridge Shank chickens after intramuscular administration, it is urgent for our further investigation of the metabolism‐based mechanism of action, ensuring the widespread use of palmatine in the animal breeding industry. Meanwhile, considering the result that palmatine has obvious DNA toxicity and a complex effect on metabolic enzymes in the liver reported in the literature (Long et al. 2019), much more attention should be given to comprehensive toxicity evaluation and toxicity identification of palmatine in Partridge Shank chickens in our subsequent research.

## Author Contributions

Jun Liang conceived the study design. Chaoxi Chen suggested a research conception. Chaoxi Chen, Yue Bu Mo A‐ga, and Xiuhua Kuang performed the experiments and collected samples. Ye Li performed HPLC‐UV for drug analysis. Chaoxi Chen and Yue Bu Mo A‐ga wrote and edited the manuscript. Rong Xiang and Jun Liang revised the final version of the manuscript. All authors analysed the data.

## Ethics Statement

The experimental procedures were performed in line with the guidelines of the Laboratory Animal Management Statute of the College of Veterinary Medicine, Henan Agricultural University and approved by the Animal Ethics Research Committee of the Faculty of Henan Agricultural University.

## Peer Review

The peer review history for this article is available at https://publons.com/publon/10.1002/vms3.70416.

## Data Availability

Apart from the processed data relating to part of an ongoing study that cannot be shared, the other data that support the findings of this study are available from the corresponding author upon reasonable request.
